# Determinants of attentional modulation near the hands

**DOI:** 10.3389/fpsyg.2013.00858

**Published:** 2013-11-18

**Authors:** Holger Schultheis, Laura A. Carlson

**Affiliations:** ^1^Department of Informatics, Universität BremenBremen, Germany; ^2^Department of Psychology, University of Notre DameNotre Dame, IN, USA

**Keywords:** embodiement, hand posture, visual search, attentional disengagement, situational determinants

## Abstract

A series of visual search experiments conducted by Abrams et al. (2008) indicates that disengagement of visual attention is slowed when the array of objects that are to be searched are close to the hands (hands on the monitor) than if they are not close to the hands (hands in the lap). These experiments establish the impact one's hands can have on visual attentional processing. In the current paper we more closely examine these two hand postures with the goal of pinpointing which characteristics are crucial for the observed differences in attentional processing. Specifically, in a set of 4 experiments we investigated additional hand postures and additional modes of response to address this goal. We replicated the original Abrams et al. (2008) effect when only the two original postures were used; however, surprisingly, the effect was extinguished with the new range of postures and response modes, and this extinction persisted across different populations (German and English students), and different experimental hardware. Furthermore, analyses indicated that it is unlikely that the extinction of the effect was caused by increased practice due to additional blocks of trials or by an increased probability that participants were able to guess the purpose of the experiment. As such our results suggest that in addition to the nature of the postures of the hand, the number of postures is a further important factor that influences the impact the hands have on visual processing.

## Introduction

In certain situations humans show an inclination to hold in their hands the target of visual perception. Regarding object perception, for example, the request “May I take a look at this?” often implicitly contains the request to be allowed to actually hold the object in one's own hands. Similarly, in reading, some people prefer to read with the text held in their hands rather than read on the computer screen, although the screen may actually provide better (perceptual) access to the text (e.g., due to adjustable size, contrast, and brightness or due to text processing tools such as full text search).

What are the reasons for such an inclination to hold in one's hands the target of visual perception? A set of recent studies suggests that one reason may be that the presence of the hands influences perceptual processes: The hands being closer to task-relevant visual stimuli modulates (a) the perceived size of objects (Vishton et al., 2007; Linkenauger et al., 2010), (b) the figure-ground assignment in the viewed stimuli (Cosman and Vecera, 2010), (c) the attentional prioritization of space (Reed et al., 2006, 2010; Davoli and Brockmole, 2012), and (d) the shifting of attention (Abrams et al., 2008; Pollux and Bourke, 2008; Davoli et al., 2012).

**Figure 1:**
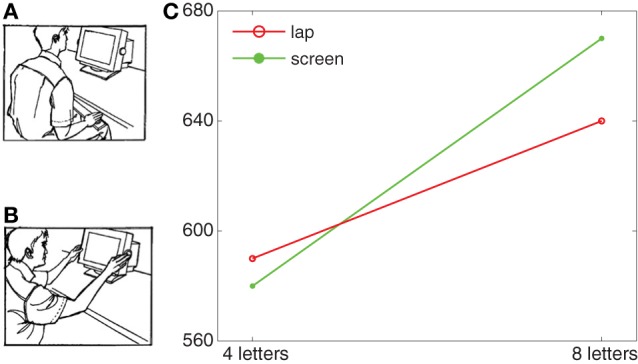
**Lap posture (A) and screen posture (B) as employed by Abrams et al. (2008) and an exemplary pattern of response time results (C).** Parts **(A)** and **(B)** reproduced from Figure 1 of Abrams et al. (2008).

Take, for instance, the shifting of attention effect observed by Abrams et al. (2008) in a number of visual search experiments. In each of these experiments participants had to search for one of two target letters and press one of two corresponding response buttons once they found the target letter. On each trial 3 or 7 distracter letters were presented together with the target letter. Across blocks of search trials the location of participants' hands was varied such that during half of the blocks the hands were in the participant's lap (Figure 1A) and during the other half of the blocks, the hands were on the monitor (Figure 1B). It is common to observe a *set size effect* in visual search, that is, an increase of the time required to correctly respond to the present target with an increase of the number of distracters presented concurrently with the target. In line with this, Abrams et al. (2008) found reliably higher response times for search when 7 distracters were shown than when 3 distracters were shown. Furthermore, this set size effect (e.g., the slope of the response time function from 3 to 7 distractors) was significantly steeper when the hands were on the monitor than when they were in the lap (e.g., steeper slope for green line than red line in Figure 1C). Based on two additional experiments examining inhibition of return and attentional blink for the two hand postures, Abrams et al. (2008) concluded that the difference between the two hand postures in visual search was due to a modulation of attentional disengagement: When the hands are on the monitor it is harder to disengage attention from a currently attended distracter to continue search for the target. This in turn gives rise to a more pronounced set size effect for the screen posture than for the lap posture.

In combination with the other studies mentioned above, the work of Abrams et al. (2008) suggests that visual processing near the hands is characterized by enhanced perception, facilitated attentional engagement, slowed attentional disengagement, and increased focus on visual detail (Brockmole et al., 2013). These modulations of visual processing are often assumed to be rooted in the involvement of bimodal visuo-tactile neuron populations in the parietal and premotor cortices (Graziano and Gross, 1993; Tseng et al., 2012).

In the current paper, we more closely examine the attentional disengagement effect of Abrams et al. (2008), seeking to identify its situational determinants. We consider the following possibilities that emerge out of a comparison of differences across the screen and lap postures: *proximity*: whether the hands are close or not close to the task-relevant stimuli; *spanning*: whether the hands “embrace” the stimuli, that is, to what extent the stimuli are between the hands; *palms*: whether the palms of the hands face toward or away from the stimuli; and *response direction*: whether the response given by the hand is a response toward the visual stimuli or away from the stimuli. Previous research has suggested that the determinants proximity (Cosman and Vecera, 2010; Adam et al., 2012), spanning (Tseng and Bridgeman, 2011), and palms (Reed et al., 2010; see also Brown et al., 2009) have an influence on attentional modulation, particularly engagement and enhanced perception. We ask here whether these factors also influence attentional disengagement as reflected within the Abrams et al. paradigm. In previous work within this paradigm, the different determinants were either confounded [e.g., proximity, spanning, and palms in Abrams et al. (2008)] or not manipulated [e.g., spanning and palms in Pollux and Bourke (2008)]. We considered response direction as an additional potential situational determinant, because the ease with which hands may act on a stimulus is directly related to whether one responds toward or away from the stimulus, while one experiment of Abrams et al. (2008) found the attentional disengagement effect also when participants responded by foot (i.e., without the hands responding toward the stimulus).

**Figure 2:**
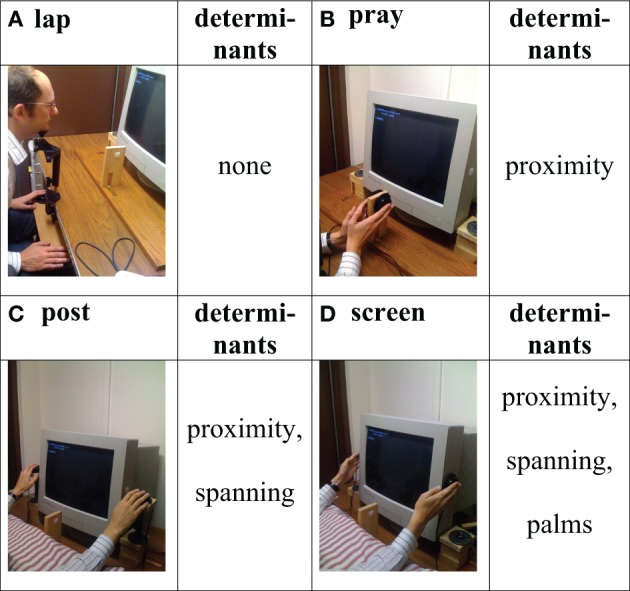
**Hand postures used in Experiment 1, labels for the hand postures, and determinants instantiated by the posture: (A)** lap posture, **(B)** pray posture, **(C)** post posture, and **(D)** screen posture.

These four situational determinants were investigated in the current experiments employing the same task as the experiments in Abrams et al. (2008). In Experiment 1, the hand postures lap and screen (Figures 1A,B, respectively), employed by Abrams et al. (2008) were complemented by new hand postures such that the postures differed in the extent to which they instantiated the determinants. In Experiment 1 we added two postures we called *pray* posture and *post* posture to yield the four postures shown in Figure 2. In the screen posture, the hands were close to the stimuli, spanned the stimuli, and the palms of the hands were facing the stimuli (i.e., instantiated proximity and spanning and palms). In the post posture, the hands spanned the monitor and were close to the screen (i.e., instantiated spanning and proximity) but the palms were not facing the stimuli. In the pray condition, the hands did not span the monitor and the palms were not facing the stimuli, but the hands were close to the screen (instantiating proximity). Finally, in the lap posture, the hands were far from the screen, did not span the monitor, and the palms did not face the stimuli (i.e., instantiated none of the factors). Accordingly, comparing the set size effects as reflected in the slopes across the different postures allowed us to examine the differential impact of the various determinants on attentional disengagement. If, for example, the set size effect was larger (i.e., a steeper slope) in the pray posture than in the lap posture, this would provide support for the idea that proximity alone contributes to a slowing of attentional disengagement. If the set size effect was larger in the screen posture than in the post posture, this would indicate that the palm determinant contributes to the slowing of disengagement in addition to the determinants proximity and spanning.

**Figure 3:**
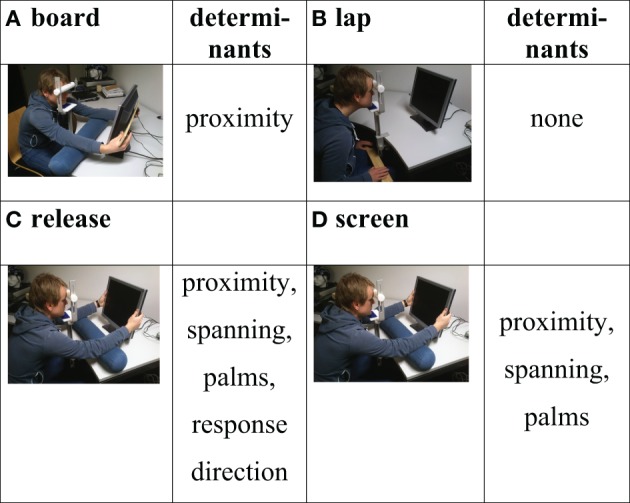
**Hand postures used in the Experiments 2–4, labels for the hand postures, and determinants instantiated by the posture: (A)** board posture, **(B)** lap posture, **(C)** release posture, and **(D)** screen posture.

As shown in Figure 3, in Experiments 2–4 we examined the original screen and lap postures, and also a *board* posture which only instantiated proximity, and a *release* posture which was identical to the screen posture, but in which participants responded by releasing the appropriate response button, that is, the release posture instantiated the same determinants as the screen posture, but additionally manipulated the response direction determinant.

## Overview of experiments

Experiments 1–4 were all extended and modified versions of the visual search experiments of Abrams et al. (2008). The common methodology was as follows. On each trial, participants performed a visual search task to find a target letter among a set of letters simultaneously displayed in random locations on the screen. The target could be one of two letters, and participants responded based on the identity of the letter, pressing one response button for one letter and the other response button for the other letter. Each visual search screen was comprised of either 4 or 8 letters, with one of these letters being a target and the other letters being distracters. As soon as participants discovered the target, they were asked to press the corresponding response button. The buttons were configured in different positions across search trials, as described further below. All experiments included the two postures originally employed in Experiment 1 of Abrams et al. (2008), that is, the *lap* posture (Figure 1A) and the *screen* posture (Figure 1B). As in Abrams et al. (2008) the response time difference between small (4 letters) and large (8 letters) search sets (e.g., the slope, see Figure 1C) was used as an index of the set size effect, and the assumption was that stronger set size effects indicate slower attentional disengagement.

Our experiments extended the original experiments by augmenting the lap and the screen postures with additional postures. In Experiment 1 we added the pray and post postures as shown in Figure 2 and described earlier. Surprisingly, Experiment 1 failed to replicate the original finding of Abrams et al. (2008), with no significant difference in the set size effects between the lap and screen postures. Therefore, in Experiments 2–4 we set out to discover why the effect became extinguished. In doing so, we employed a physical set up (see Figure 3) that was more similar to that of Abrams et al. (2008). In Experiment 2, we included only the lap (Figure 3B) and screen postures (Figure 3D) so as to directly replicate Abrams et al. (2008). Since replication was successful in Experiment 2, Experiment 3 was designed to investigate whether the replication failure in Experiment 1 was due to the physical setup or the addition of extra postures. Accordingly, Experiment 3 added the board (Figure 3A) and release (Figure 3C) postures to the lap and screen postures. Interestingly, Experiment 3 again failed to replicate the originally found difference between the lap and screen postures. This leads us to believe that the extinction of the original effect was likely caused by the addition of extra postures. In Experiment 4 we asked how sensitive the original effect is to the addition of postures by adding only a single extra posture, the release posture, to the lap and screen postures. The fact that Experiment 4 failed to replicate the original effect provided additional support to the idea that the extinction of the effect is caused by adding extra postures to the original setup.

## Experiment 1

In Experiment 1, the four postures employed were lap, pray, post, and screen. As indicated in Figure 2, these four postures differed in the extent to which the determinants proximity, spanning, and palms were instantiated when participants placed their hands on the buttons to respond during the visual search task. Accordingly, if proximity contributed to the slowing of attentional disengagement effected by the hands, one would expect a difference in the set size effects between the lap and the pray posture. If spanning had a substantial impact on the slowing of attentional disengagement, this should show up as a difference in the set size effects between the pray and the post posture, and if palms influenced attentional disengagement, one should observe a difference in the set size effects between the post and the screen posture.

### Methods

#### Participants

Sixty-two University of Notre Dame undergraduates participated in Experiment 1. They were compensated by partial credit for an undergraduate psychology course. All participants in this and subsequent experiments gave informed consent.

#### Materials and apparatus

Participants sat facing a 20″ CRT monitor with their chins in a chinrest. The chair and chinrest were adjusted for each participant such that their eyes were vertically and horizontally aligned with the center of the monitor. Following Abrams et al. (2008), each visual search set contained one target letter and 3 or 7 distracter letters. The target letter was either an “S” or an “H” and all letters were 3° high and 1.5° wide. Search sets were presented in a display area that measured 33° of visual angle wide and 21° of visual angle high, centered on the monitor's center. The location of the letters was determined randomly, subject to the constraint that any two letters were at least 0.75° apart. In contrast to Abrams et al. (2008), we employed “R” and “T” (instead of “U” and “E”) as distracter letters to avoid the possibility that the randomly placed target and distracters might spell anything meaningful. Each distracter was randomly determined to be either an “R” or a “T.” Response buttons were 6 cm in diameter and were attached so as to configure different postures across trials, as described above. The buttons were connected to the computer through a modified keyboard such that pressing the buttons produced the characters “/” and “z,” respectively.

The distance between the two hands was 35, 5, 60, and 53 cm in the lap, pray, post, and screen postures, respectively. The distance of the hands to the monitor was 50, 10, and 3.5 cm in the lap, pray, and post postures, respectively. The distance between the viewer and the hands was 30 cm in the pray posture and 40 cm in the post and screen postures.

#### Procedure

At the start of each trial a fixation cross (1.5° × 1.5°) was shown at the center of the display area. After 500 ms the fixation cross was replaced with the search set. Participants were instructed to identify which of the two target letters was present, and to indicate its identity by pressing the corresponding response button. The mapping of target letters to response buttons was counterbalanced across subjects. Once a button was pressed, the participant received feedback when the response was faster than 100 ms (“Too fast!”), slower than 1500 ms (“Too slow!”), and/or the wrong button was pressed (“Wrong key pressed!”). The inter-trial-interval was 2000 ms.

Overall, each participant performed 256 trials that were presented in a set of 4 blocks of 64 trials each. For each block the hands were in a different posture, with the order of postures counterbalanced across subjects. Of the 64 trials in each block, there were 16 replications of all possible target letter-set size (S-setsize 4; H-setsize 4; S-setsize 8; H-setsize 8) combinations.

### Results

A 0.05 level of significance was adopted for conventional statistical analyses in this and all following experiments. The conventional analysis was complemented by a Bayesian analysis as described in Masson (2011; see also Wagenmakers, 2007). This analysis provided the probability *P*_BIC_(H_0_|D) that the null hypothesis, *H*_0_, was true given the available set of data *D*. The probability of the alternative hypothesis *H*_1_ can be computed as *P*_BIC_(H_1_ |D) = 1 − P_BIC_(H_0_ |D). To indicate the support the experimental results lend to *H*_0_ and *H*_1_, respectively, the probabilities *P*_BIC_(H_0_ |D) are reported in addition to the results of the conventional analysis. Since accuracies were often very close to 100%, they were arcsine-transformed before statistical analyses.

#### Response times

Trials in which participants answered incorrectly were excluded from response time analyses. Furthermore, standard deviations and means were computed for each individual and condition and response times outside a 2.5 * *SD* range from the mean were excluded from analyses^1^. Overall 7% of all trials were excluded from analyses.

**Figure 4:**
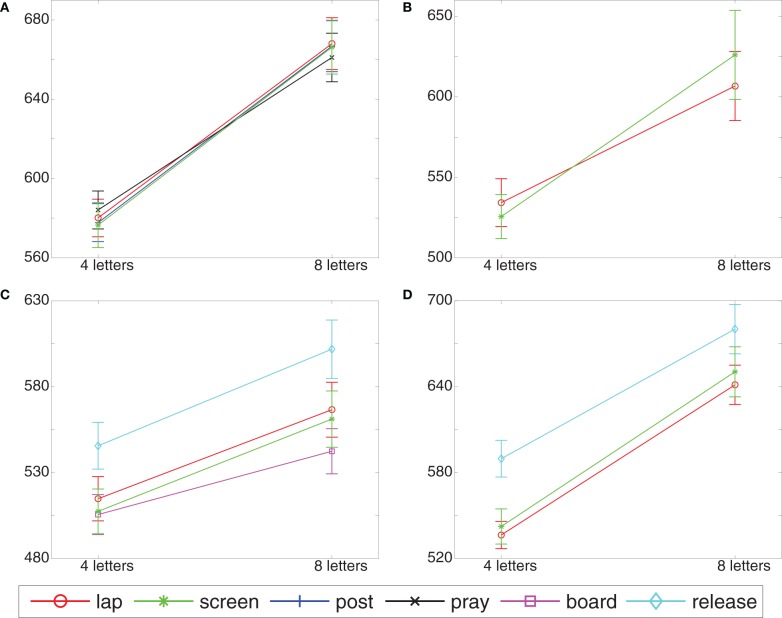
**Response time results for Experiment 1 (A), Experiment 2 (B), Experiment 3 (C), and Experiment 4 (D)**.

Figure 4A and Table 1 show the mean response times and the set size effects, respectively, for the four postures. A 4 (posture) × 2 (set size) analysis of variance revealed that search was faster in small (*M* = 568 ms) than in large (*M* = 656 ms) sets [*F*_(1, 61)_ = 332.2, *p* < 0.001; *P*_BIC_(H_0_|D) < 10^−25^]. Posture had no clear effect on search speed [*M*s = 613, 612, 614, and 609 ms for lap, post, pray, and screen postures, respectively; *F* < 1; *P*_BIC_(H_0_ |D) = 0.92] nor on set size effects [mean response time differences between set size 8 and set size 4 were 87, 90, 80, and 95 ms for lap, post, pray, and screen, respectively; *F* < 1, *P*_BIC_(H_0_ |D) = 0.79]. In particular there was no significant difference in set size effects between lap and screen postures [*F* < 1, *P*_BIC_(H_0_ |D) = 0.85].

**Table 1:** **Mean set size differences (mean response times for setsize 8–setsize 4) and corresponding standard errors in parentheses for the postures of Experiments 1–4**.

	**Postures**			
Experiment 1	**Lap**	**Screen**	**Pray**	**Post**
	87 (7.5)	95 (7.4)	80 (6.7)	90 (8.7)
Experiment 2	Lap^*^	Screen^*^		
	72 (10.1)	100 (16.4)		
Experiment 3	**Lap**	**Screen**	**Release**	**Board**
	48 (5.6)	61 (7.9)	50 (7.5)	41 (5.1)
Experiment 4	**Lap**	**Screen**	**Release**	
	105 (7.1)	108 (9.5)	91 (8.6)	

* *significant posture difference*.

#### Practice effects

To our surprise, Experiment 1 did not replicate the difference between set size effects in the lap and screen postures originally reported by Abrams et al. (2008). One potential cause for the failure to replicate are practice effects that might have been brought about by including two additional postures. Assuming that the participants' skill in performing the visual search task increases with the duration of performing the search task, adding extra postures might have given rise to more skilled visual search performance. If more skilled performance is not subject to the same attentional modulation as less skilled performance, an elimination of the attentional disengagement effect found by Abrams et al. (2008) could have resulted. Accordingly, adding extra postures may have lead to practice effects that eliminate differences in attentional disengagement between postures.

To assess the existence of practice effects and their potential impact on attentional disengagement, we conducted a set of extra analyses for this and all following experiments. The first set of analyses collapse across the specific postures and consider search performance by block instead. Replacing the factor posture by the factor block allowed examination of how search speed and set size effects depend on the block number, that is, on how much practice participants had already gained in the visual search task. The second set of analyses compares two groups of participants^2^. Group 1 consists of participants that experienced the lap and screen postures in the first two blocks; group 2 consists of participants that experienced the lap and screen postures in the last two blocks.

A 4 (block) × 2 (set size) analysis of variance revealed that search got faster with increasing practice [651, 613, 597, and 587 ms for blocks 1, 2, 3, and 4, respectively; *F*_(3, 183)_ = 21.6441, *p* < 0.001, *P*_BIC_(H_0_ |D) < 7.5 * 10^−12^]. Furthermore, a significant block × set size interaction indicated that the set size effect decreased with practice [103, 89, 79, 77 ms for blocks 1, 2, 3, and 4, respectively; *F*_(3, 183)_ = 3.4754, *p* < 0.05, *P*_BIC_(H_0_ |D) = 0.073]. More specifically, the overall interaction was largely driven by significant differences in set size effects between block 1 and block 3 [*F*_(1, 61)_ = 6.1683, *p* < 0.05, *P*_BIC_(H_0_ |D) = 0.28] as well as block 1 and block 4 [*F*_(1, 61)_ = 7.9136, *p* < 0.01, *P*_BIC_(H_0_ |D) = 0.15].

A 2 (posture) × 2 (set size) × 2 (presentation time) analysis of variance with the first two factors within subjects and the third factor between subjects also indicated that search got faster with increasing practice [652 ms and 597 ms for participants experiencing lap and screen in the first and last blocks, respectively; *F*_(1, 20)_ = 4.778, *p* < 0.05, *P*_BIC_(H_0_ |D) = 0.308]. However, the lack of any significant interaction involving presentation time suggests that practice had no clear effect on the differences in the magnitude of the set size effect between the lap and screen postures.

Nevertheless, the first type of practice analysis indicates an impact of practice on the magnitude of the set size effect and, accordingly, raises the possibility that the failure to replicate the original difference in set size effects between lap and screen postures is due to practice effects. To further investigate this possibility, we re-analyzed the response times considering only data from the lap and screen postures of those participants that experienced these postures in the first two blocks (*N* = 12). The resulting 2 (posture) × 2 (set size) analysis of variance exhibited no clear indication for differences in set size effects for lap (94 ms) and screen (104 ms) postures [*F* < 1; *P*_BIC_(H_0_ |D) = 0.76]. Consequently, although practice had an impact on the magnitude of set size effects, our results did not find any evidence that these practice effects were responsible for not replicating the original finding of Abrams et al. (2008).

#### Accuracies

Table 2 shows the mean accuracies for the four postures. Accuracies were generally high, ranging from 82% to 100% with a mean accuracy of 96%. A 4 (posture) × 2 (set size) analysis of variance revealed no effect of posture [*F*_(3, 183)_ = 1.8257, *p* > 0.14; *P*_BIC_(H_0_ |D) = 0.47] and no interaction [*F*_(3, 183)_ = 1.1161, *p* > 0.34; *P*_BIC_(H_0_ |D) = 0.72]. The main effect of set size was significant [*F*_(1, 61)_ = 4.1335, *p* < 0.05; *P*_BIC_(H_0_ |D) = 0.51] indicating lower accuracy for search in large sets (95.6%) than in small sets (96.2%).

**Table 2:** **Mean percent correct and corresponding standard errors in parentheses for the conditions of Experiments 1–4**.

	**Postures**				
Experiment 1	**Set size**	**Lap**	**Screen**	**Pray**	**Post**
	large	95.1 (0.72)	95.1 (0.66)	95.9 (0.66)	96.2 (0.6)
	small	96 (0.59)	95.7 (0.72)	97 (0.46)	96.1 (0.52)
Experiment 2	**Set size**	**Lap**	**Screen**		
	large	95.2 (0.97)	96 (0.82)		
	small	96.7 (0.6)	96.7 (0.76)		
Experiment 3	**Set size**	**Lap**	**Screen**	**Release**	**Board**
	large	96.3 (0.74)	96.3 (0.63)	96.4 (0.89)	96 (0.7)
	small	96.8 (0.61)	96.6 (0.61)	97 (0.62)	96 (0.56)
Experiment 4	**Set size**	**Lap**	**Screen**	**Release**	
	large	96.4 (0.73)	96.2 (0.68)	96.4 (0.55)	
	small	95.7 (0.75)	96.9 (0.51)	96.8 (0.71)	

#### Practice effects

As for response times, we analyzed accuracies for practice effects. A main effect of block indicated that participants performed more accurately with increasing numbers of blocks [95.4, 95.2, 96, and 96.9% for blocks 1, 2, 3, and 4, respectively; *F*_(3, 183)_ = 6.4875, *p* < 0.001, *P*_BIC_(H_0_ |D) = 0.0011]. Practice had, however, no significant influence on the magnitude of the set size effect [*F* < 1; *P*_BIC_(H_0_ |D) = 0.82]. Similarly, the second type of practice analyses revealed a significant increase in performance with practice [*F*_(1, 20)_ = 6.503, *p* < 0.05; *P*_BIC_(H_0_ |D) = 0.174].

### Discussion

The observed response times and accuracies gave no indication that attentional disengagement was different for the different postures. Moreover, the lap and screen postures, which lead to significantly different set size effects in Experiment 1 of Abrams et al. (2008) showed no clear sign of differential attentional disengagement. This failure to replicate the originally reported difference between the lap and screen condition renders it problematic to interpret the presence or absence of differences in attentional disengagement between all postures.

Accordingly, one approach might be to restrict analyses of the influence of the different determinants to those participants that show the disengagement effect reported by Abrams et al. (2008), that is, those participants that exhibit a stronger set size effect in the screen condition than in the lap condition. The logic here would be something like: Given that this subset of participants showed a difference in the screen and lap conditions, how then do their other conditions compare? While this logic is sound, such a split may be misleading, however, because it may artificially introduce significant effects between postures. To see this, assume a situation in which the hand posture has **no** influence on the measured response time differences. Due to noise, set size effects will rarely ever be exactly identical across the four postures. Therefore, each participant can be categorized as showing one of 24 (4!) possible orders of the postures in terms of the magnitudes of the set size effect. For example, a participant may exhibit the order lap < pray < post < screen and another may exhibit the order pray < screen < lap < post. For half of all possible orders the relation lap < screen holds. Selecting replicators therefore, amounts to reducing the set of possible orders from 24 to 12. This is problematic, because the relations pray < screen, post < screen, lap < pray, and lap < post each occur in 8 of the 12 orders. Accordingly, selecting replicators may introduce a bias to find evidence for these orderings, even if actually no systematic differences exist between postures. Therefore, an analysis restricted to replicators cannot remedy the problem of a missing difference between lap and screen postures. We are left to conclude that our first experiment was unsuccessful in examining the differential impact of proximity, spanning, and palms on attentional disengagement.

We note that our failure to replicate the difference across the screen and lap postures was not due to a lack of statistical power. The Bayesian analyses revealed that the posterior probability for the *H*_0_ (no differences in set size effects between postures) was considerably higher than the posterior probability of the *H*_1_. Put differently, given the posterior probabilities for the two hypotheses, the experimental results provided positive support (see Masson, 2011) for a lack of an effect of posture on attentional disengagement. Furthermore, although additional analyses indicated the presence of practice effects, we found no clear evidence that these practice effects were responsible for our failure to replicate.

Accordingly, Experiment 2 sought to uncover why we failed to replicate the basic screen and lap posture difference observed robustly across several studies (Abrams et al., 2008; Davoli and Abrams, 2009; Tseng and Bridgeman, 2011). Two aspects of our experimental setup seemed likely candidates. First, in contrast to the study of Abrams et al. (2008), our experiment employed 4 instead of 2 button postures. Second, although the experimental task was the same as in Abrams et al. (2008), there were several differences in operationalization. Specifically, our experiment employed a bigger monitor (20″) than the original study (18″); we used 64 trials per block and the fixation cross disappeared with the onset of the search array, whereas Abrams et al. (2008) used 128 trials per block and the fixation cross remained on the screen during search. Finally, in order to implement the pray and post postures we needed to add wooden constructions to allow us to position the response buttons in the appropriate locations; no such constructions were present in the visual search experiments of Abrams et al. (2008). To investigate the extent to which these differences were responsible for the elimination of the original effect our second experiment employed only the lap and screen postures and reduced the differences in operationalization.

## Experiment 2

The goal of Experiment 2 was to replicate the findings of Abrams et al. (2008), using a physical setup as similar as possible to the original setup. Specifically, we employed the lap and screen postures as shown in Figures 3B,D, we replaced the 20″ CRT monitor by a 19″ TFT monitor, increased the number of trials from 64 trials per block to 128 trials per block and removed the wooden constructions that were necessary for the pray and post conditions.

### Methods

#### Participants

Twenty-four University of Bremen undergraduates participated in Experiment 2. They received a monetary compensation for their participation.

#### Materials and apparatus

The 20″ CRT monitor was replaced by a 19″ TFT monitor. Response buttons were 3 cm in diameter and were connected to the computer as an additional keyboard device such that pressing the buttons produced the characters “/” and “z,” respectively. All other materials and apparatus were identical to those in Experiment 1.

In this setup, the distance between the two hands was 30 cm and 31 cm in the lap and screen postures, respectively. The distance of the hands to the monitor was 45 cm in the lap posture and the distance between the viewer and the hands was also 45 cm in the screen posture.

#### Procedure

Trials proceeded as described for Experiment 1 with the exception that the fixation cross remained on the screen during the whole trial. Overall, each participant performed 256 trials that were presented in blocks of 128 trials. For each block the hands were in a different posture such that both of the postures lap and screen (see Figure 3) were used for exactly one block. The order of postures was counterbalanced across subjects.

### Results

#### Response times

Trials in which participants answered incorrectly were excluded from response time analyses. Furthermore, standard deviations and means were computed for each individual and condition and response times outside a 2.5 * *SD* range from the mean were excluded from analyses. Overall 7% of all trials were excluded from analyses.

Figure 4B and Table 1 show the mean response times and the set size effects, respectively, for the two postures. A 2 (posture) × 2 (set size) analysis of variance revealed that search was faster in small (*M* = 530 ms) than in large (*M* = 616 ms) sets [*F*_(1, 23)_ = 49.162, *p* < 0.001; *P*_BIC_(H_0_ |D) < 10^−6^]. Furthermore, the significant posture × size interaction [*F*_(1, 23)_ = 5.9, *p* < 0.05; *P*_BIC_(H_0_ |D) = 0.24] indicated that the set size effect was smaller for the lap posture (mean response time difference of 72 ms) than the set size effect for the screen posture (mean response time difference of 100 ms). There was no significant main effect of posture on response times [*F* < 1; *P*_BIC_(H_0_ |D) = 0.812].

#### Practice effects

There were no clear practice effects: neither the main effect of block [*F*_(1, 23)_ = 1.9575, *p* > 0.15; *P*_BIC_(H_0_ |D) = 0.65] nor the block × set size interaction [*F* < 1; *P*_BIC_(H_0_ |D) = 0.75] were significant.

#### Accuracies

Table 2 shows the mean accuracies for the lap and the screen posture. Accuracies ranged from 84% to 100% with a mean accuracy of 96%. A 2 (posture) × 2 (set size) analysis of variance revealed no effect of posture [*F*_(1, 23)_ = 2.757; *P*_BIC_(H_0_ |D) = 0.557] and no interaction [*F* < 1; *P*_BIC_(H_0_ |D) = 0.83]. The main effect of size approached significance [*F*_(1, 23)_ = 4.007, *p* < 0.06; *P*_BIC_(H_0_ |D) = 0.416] indicating a slightly lower accuracy for search in large sets (95.6%) than for search in small sets (96.7%).

#### Practice effects

There were no clear practice effects: neither the main effect of block [*F* < 1; *P*_BIC_(H_0_ |D) = 0.82] nor the block × set size interaction [*F* < 1; *P*_BIC_(H_0_ |D) = 0.81] were significant.

### Discussion

The results of Experiment 2 replicate Abrams et al. (2008), using 2 postures and an operationalization of the procedure that closely mimicked the original study. The aim of Experiment 3 was thus, to examine whether the failure to replicate the original effect in Experiment 1 was due to its inclusion of an increased number of postures. Accordingly, we used the operationalization of the procedure from Experiment 2 but added two postures to the lap and screen postures. The extra postures, called board (Figure 3A) and release (Figure 3C), were chosen such that they did not require any additional hardware constructions. As a result, though employing two extra postures, the physical setup in Experiment 3 was the same as in Experiment 2 (see Figure 3); in addition, the procedural changes (e.g., fixation cross present through the trial; 128 trials per block) were as in Experiment 2. If the addition of these postures leads to the extinction of the effect, this would provide evidence that the number of postures was responsible for the failure to replicate. If, in contrast, the original effect is replicated, the postures board and release would allow investigating the impact of the determinants of proximity and response direction. If proximity is of importance, there should be a significantly increased set size effect in the board posture compared to the lap posture. If response direction influences attentional disengagement, there should be a larger set size effect in the screen posture than the release posture.

## Experiment 3

Experiment 3 employed the four postures board, lap, release, and screen as shown in Figures 3A–D, respectively, but otherwise employed the same physical setup and operationalization as Experiment 2.

### Methods

#### Participants

Twenty-four University of Notre Dame undergraduates participated in Experiment 3. They were compensated by partial credit for an undergraduate psychology course.

#### Materials and apparatus

Materials and Apparatus were the same as in Experiment 2, but distances differed slightly. The distance between the two hands was 37 cm for the screen and release postures and 40 cm for the board and lap postures. The distance of the hands to the monitor was 60 cm in the lap posture and 4 cm in the board posture. The distance between the viewer and the hands was 39 cm in the board posture and 35 cm in the screen and release postures.

#### Procedure

Trials proceeded as described for Experiment 2. Overall, each participant performed 512 trials that were presented in blocks of 128 trials. For each block the hands were in a different posture such that each of the four postures board, lap, release, and screen (see Figure 3) was used for exactly one block. The order of postures was counterbalanced across subjects.

### Results

#### Response times

Trials in which participants answered incorrectly were excluded from response time analyses. Furthermore, standard deviations and means were computed for each individual and condition and response times outside a 2.5 * *SD* range from the mean were excluded from analyses. Overall 6% of all trials were excluded from analyses.

Figure 4C and Table 1 show the mean response times and set size differences, respectively, for the four postures. A 4 (posture) × 2 (set size) analysis of variance revealed that search was faster in small (*M* = 521 ms) than in large (*M* = 571 ms) sets [*F*_(1, 23)_ = 0.131.93, *p* < 0.001; *P*_BIC_(H_0_ |D) < 10^−9^] and that responses were slower in the release posture than in the other postures [*M*s = 525, 542, 581, and 535 ms for board, lap, release, and screen postures, respectively; *F*_(3, 69)_ = 17.95, *p* < 0.001; *P*_BIC_(H_0_ |D) < 10^−8^]. However, there was no significant interaction [*F*_(3, 69)_ = 1.96, *p* > 0.1, *P*_BIC_(H_0_ |D) = 0.308], suggesting that the set size effect was not mediated by posture, (*M*s = 41, 48, 50, and 61 ms for board, lap, release, and screen, respectively). In particular, the set size effects in the lap and the screen postures did not differ significantly [*F*_(1, 23)_ = 2.35, *p* > 0.1, *P*_BIC(H_0_ |D) = 0.603_].

#### Practice effects

A 4 (block) × 2 (set size) analysis of variance revealed that search got faster with increasing practice [565, 544, 544, and 529 ms for blocks 1, 2, 3, and 4, respectively; *F*_(3, 69)_ = 4.5517, *p* < 0.01, *P*_BIC_(H_0_ |D) = 0.013]. The interaction did not reach significance [*F* < 1, *P*_BIC_(H_0_ |D) = 0.809], indicating that practice had no clear impact on the magnitude of the set size effect. A 2 (posture) × 2 (set size) × 2 (presentation time) analysis of variance found no evidence for increased search speed with practice [*F* < 1; *P*_BIC_(H_0_ |D) = 0.703] nor any significant interaction involving presentation time.

#### Accuracies

Table 2 shows the mean accuracies for the four postures. Accuracies were again high, ranging from 90% to 99% with a mean accuracy of 96%. A 4 (posture) × 2 (set size) analysis of variance revealed no effect of posture [*F* < 1; *P*_BIC_(H_0_ |D) = 0.685], no effect of size [*F* < 1; *P*_BIC_(H_0_ |D) = 0.83], and no significant interaction [*F* < 1; *P*_BIC_(H_0_ |D) = 0.849].

#### Practice effects

There were no clear practice effects: neither the main effect of block [*F*_(3, 69)_ = 1.6538, *p* > 0.15; *P*_BIC_(H_0_ |D) = 0.41] nor the block × set size interaction [*F* < 1; *P*_BIC_(H_0_ |D) = 0.734] were significant. Similarly, the second set of practice analyses yielded no indication of an influence of practice on accuracy [*F* < 1; *P*_BIC_(H_0_ |D) = 0.739] or on the magnitude of the set size effect (no interaction involving presentation time reached significance).

### Discussion

Despite the increased similarity of the experimental setup to the original study of Abrams et al. (2008), results largely mirrored those of Experiment 1. In particular, there was no clear indication of a differential effect of posture on attentional disengagement—neither across all postures nor when only comparing lap and screen postures. Furthermore, analyses render it unlikely that the lack of a disengagement effect is due to practice effects. The only difference in results between Experiment 1 and Experiment 3 is the main effect of posture that arose from slowed responding in the release posture. Based on personal experience running through the experiment and spontaneous comments by participants, this slowing may have been due to the unfamiliar response mode of releasing instead of pressing the response buttons.

In the light of these results and the results of the previous two experiments, it seemed more likely that the absence of an attentional disengagement effect was due to the increased number of postures than due to dissimilarities in experimental setups. To further assess the sensitivity of the disengagement effect to the number of additional postures, we employed only one extra posture, the release posture, in Experiment 4. The release posture was chosen because this posture allowed using the same button configurations as in Experiment 2 (thus, rendering the setup of Experiment 4 identical to the setup of Experiment 2) while also adding a new response direction. In addition, Experiments 1 and 3, which failed to replicate were run at the University of Notre Dame while the first author was visiting during a research stay, and Experiment 2 was run at the University of Bremen. Thus, being run at the University of Bremen, Experiment 4 enabled us to check whether the failure to replicate in Experiments 1 and 3 was due to population differences.

## Experiment 4

### Method

Materials, apparatus, and procedure were identical to those in Experiment 2 and 3. Distances were the same as in Experiment 2.

Thirty University of Bremen undergraduates participated in Experiment 4. They chose to receive either monetary compensation or partial credit for an undergraduate psychology course for their participation.

### Results

#### Response times

Trials in which participants answered incorrectly were excluded from response time analyses. Furthermore, standard deviations and means were computed for each individual and condition and response times outside a 2.5 * *SD* range from the mean were excluded from analyses. Overall 6% of all trials were excluded from analyses.

Figure 4D and Table 1 show the mean response times and the set size effects, respectively, for the three postures. As can be seen from the figure, the results largely mirrored those of Experiment 3. A 3 (posture) × 2 (set size) analysis of variance revealed that search was faster in small (556 ms) than in large (657 ms) sets [*F*_(1, 27)_ = 200.62, *p* < 0.001; *P*_BIC_(H_0_ |D) < 10^−13^]. Furthermore, response speed differed significantly across postures [*F*_(2, 58)_ = 11.716, *p* < 0.001; *P*_BIC_(H_0_ |D) < 0.001] with responses in the release posture being considerably slower (635 ms) than responses in the lap (589 ms) and the screen (596 ms) posture. However, there was no significant interaction, indicating that posture did not mediate the set size effect (*M* set sizes = 105, 91, and 108 ms for lap, release, and screen postures, respectively), either for all three postures [*F*_(2, 58)_ = 2.588, *p* > 0.08; *P*_BIC_(H_0_ |D) = 0.373] or when only considering postures lap and screen [*F* < 1; *P*_BIC(H_0_ |D) = 0.834_].

#### Practice effects

A 3 (block) × 2 (set size) analysis of variance indicated no clear main effect of block [*F* < 1; *P*_BIC_(H_0_ |D) = 0.784] and no significant block × set size interaction [*F*_(2, 58)_ = 2.48, *p* > 0.09; *P*_BIC_(H_0_ |D) = 0.398]. The 2 (posture) × 2 (set size) × 2 (presentation time) analysis of variance also did not yield any significant effect of practice on search speed [*F* < 1; *P*_BIC_(H_0_ |D) = 0.807] or the magnitude of the set size effect (no interaction including the factor presentation time reached significance).

#### Accuracies

Table 2 shows the mean accuracies for the three postures. Accuracies ranged from 85% to 99% with a mean accuracy of 96%. A 3 (posture) × 2 (set size) analysis of variance revealed no effect of either posture [*F* < 1; *P*_BIC_(H_0_ |D) = 0.884] or set size [*F* < 1; *P*_BIC_(H_0_ |D) = 0.837]. The interaction also did not reach significance [*F*_(2, 58)_ = 2.097, *p* > 0.1; *P*_BIC_(H_0_ |D) = 0.49].

#### Practice effects

A 3 (block) × 2 (set size) analysis of variance revealed that participants performed more accurately with increasing practice [95.8, 96.4, and 97% for blocks 1, 2, and 3, respectively; *F*_(2, 58)_ = 8.7924, *p* < 0.001; *P*_BIC_(H_0_ |D) = 0.003]. The interaction did not reach significance [*F*_(2, 58)_ = 1.933, *p* > 0.15; *P*_BIC_(H_0_ |D) = 0.528], indicating that practice had no clear impact on the magnitude of the set size effect. As for response times, a 2 (posture) × 2 (set size) × 2 (presentation time) analysis of variance did not indicate any substantial practice effects: Neither the main effect of presentation time [*F* < 1; *P*_BIC_(H_0_ |D) = 0.786] nor any of its interactions reached significance.

### Discussion

As in Experiments 1 and 3, there was no clear indication of a differential modulation of the disengagement of attention depending on posture, even when practice effects were taken into account. This suggests that even a single additional posture was enough to eliminate the disengagement effect reported in Abrams et al. (2008) and replicated in Experiment 2. Furthermore, since the extinction of the original effect occurred across two different sites (University of Notre Dame and University of Bremen), it is unlikely that the failure/success of replication is due to population differences.

## General discussion

Although we were able to replicate the modulation of the disengagement of attention by different hand postures originally reported by Abrams et al. (2008) in one of our experiments (Experiment 2), we found no evidence of such a modulation in the other three experiments we conducted: Whenever the experimental design included more than the originally employed lap and screen postures, the disengagement effect disappeared (Experiments 1, 3, and 4). Given the properties of our experiments it seems unlikely that the disappearance of the modulation is due to (a) the particular postures added to the lap and screen postures (pray, Figure 2B, and post, Figure 2C, were used in Experiment 1, board, Figure 3A, and release, Figure 3C, postures were used in Experiment 3 and 4), (b) the population under investigation (Experiment 1 and 3 drew on US students at the University of Notre Dame, while Experiment 4 drew on German students at the University of Bremen), or (c) the physical setup of the experiments (compare setups of Experiment 1, Figure 2, vs. setups of Experiments 3 and 4, Figure 3). Furthermore, Bayesian analyses ruled out the possibility that the failure to find an attentional disengagement effect in Experiments 1, 3, and 4 is a result of a lack in statistical power: In all three experiments the a-posteriori probability of the null hypothesis that there is no difference in attentional disengagement between the lap and screen postures is considerably higher than the probability of the alternative hypothesis [*P*_BIC_(H_0_ |D) = 0.85, 0.603, and 0.834 for Experiments 1, 3, and 4, respectively]. As an additional test we analyzed the pooled data of Experiments 3 and 4 for the postures lap, release, and screen. Again, there was no evidence of an attentional disengagement effect; neither when comparing response time differences for the lap (*M* = 80 ms) and the screen (*M* = 87 ms) postures [*F*_(1, 53)_ = 1.6834, *p* > 0.2; *P*_BIC_(H_0_ |D) = 0.76] nor when comparing accuracy differences for the lap (0.14%) and the screen (−0.55%) postures [*F*_(1, 53)_ = 1.955, *p* > 0.15; *P*_BIC_(H_0_ |D) = 0.734].

This leaves the number of postures as the most credible cause for the disappearance of the attentional disengagement effect, such that the addition of an extra posture eliminated the effect observed across the screen and lap conditions. It is, however, currently unclear why this addition leads to the extinction of the effect.

The setup of the four experiments and analyses results rule out a number of explanations. First, it seems unlikely that the disappearance of the effect is due to an increase in the number of trials that results from an increase in the number of postures. In Experiment 1, participants worked on 64 search trials for each of the four postures and, thus, participants performed 256 search trials overall. The same number of search trials was performed by participants in Experiment 2, because for each of the two postures employed in this experiment, participants completed 128 trials. Accordingly, the difference in replication between the two experiments cannot be due to the overall number of search trials.

Second, results suggest that practice does not play a major role in the disappearance of the disengagement effect. Although practice led to reduced set size effects in Experiments 1, we found no evidence that this decrease caused the extinction of the disengagement effect.

Third, it is unlikely that the extinction of the effect is brought about by an increased likelihood of guessing the purpose of the experiment due to experiencing more postures. If experiencing more postures were responsible for the disappearance of an otherwise present effect, the effect should be detectable when analyzing only the data from the lap and screen postures of those participants that experienced these postures in the first two blocks. However, conducting such analyses revealed no clear evidence for an influence of hand postures on the set size effect.

Although the sequence of experiments and their results were not as we initially anticipated, they provide an interesting and novel answer to the question that motivated our work: “What are the situational determinants for attentional disengagement?” Our results indicate that it is not only the nature of hand postures, but also the number of postures that impacts the presence or absence of attentional disengagement. That is, the number of postures itself constitutes one of the situational determinants of the attentional disengagement effect. This is in line with the fact that previous studies on attentional disengagement did not employ more than two hand postures (Abrams et al., 2008; Pollux and Bourke, 2008; Davoli et al., 2012).

Against this background it seems interesting to consider whether this situational determinant (number of postures) is specific to attentional disengagement or whether it may more generally impact a wider range of modulations of visual processing. In fact, some experiments (Reed et al., 2006, 2010; Experiment 1 of Davoli and Brockmole, 2012; Experiment 4 of Dufour and Touzalin, 2008) found an effect of the hands on attentional prioritization although they employed more than two hand postures. Accordingly, while more than two hand postures led to an elimination of the attentional disengagement effect in the current work, an increased number of postures had no comparable effect on attentional prioritization—at least not in those experiments reported in the literature. To what extent the number of postures constitutes a critical determinant for other effects the hands can have on visual processing is currently hard to judge, because the majority of previous experiments do not employ more than two hand postures within participants (Vishton et al., 2007; Cosman and Vecera, 2010; Linkenauger et al., 2010; Tseng and Bridgeman, 2011; Experiments 1–3 in Dufour and Touzalin, 2008; Experiment 2 in Davoli et al., 2010; Davoli and Brockmole, 2012; Gozli et al., 2012).

In conclusion, our experiments highlight the potential importance of the number of postures as another crucial situational determinant for the impact one's hands have on various aspects of visual processing. Given that previous studies have not systematically investigated this determinant suggests that its importance may have been underestimated so far. Our results stress the necessity to explicitly consider this factor and future work is required to examine to what extent the number of postures also influences the other effects that hands may have on visual processing.

## Author note

Holger Schultheis, Department of Informatics, Universität Bremen, Germany and Laura A. Carlson, Department of Psychology, University of Notre Dame, USA. The reported work was conducted in the scope of the project R1-[ImageSpace] of the Collaborative Research Center SFB/TR 8 Spatial Cognition. Funding by the German Research Foundation (DFG) is gratefully acknowledged. We also thank the Department of Psychology for supporting a research visit by the first author to the University of Notre Dame. Part of this research has been presented at the 32nd annual meeting of the Cognitive Science Society. We thank Richard Abrams and Christopher Davoli for helpful discussions on experimental design and Richard Abrams also for his comments on an earlier version of this manuscript. We also thank Chistopher Galeucia for his help in running Experiment 3. Correspondence concerning this article should be addressed to Holger Schultheis, Department of Informatics, Universität Bremen, Enrique-Schmidt-Str. 5, 28359 Bremen, Germany (email: schulth@informatik.uni-bremen.de).

### Conflict of interest statement

The authors declare that the research was conducted in the absence of any commercial or financial relationships that could be construed as a potential conflict of interest.

## Footnotes

^1^To check to what extent our results depend on the exclusion criterion we also analyzed response times after only excluding times that were faster than 100 ms or slower than 1500 ms. These analyses yielded the same pattern of significances as the analyses reported in the paper.

^2^We thank one of the reviewers for suggesting this additional analysis.
